# Non-Antimicrobial Drugs: Etodolac as a Possible Antimicrobial or Adjuvant Agent Against ESKAPE Pathogens

**DOI:** 10.2174/1874285801812010288

**Published:** 2018-08-31

**Authors:** Sónia G. Pereira, Vanessa S. Domingues, João Theriága, Maria de Jesus Chasqueira, Paulo Paixão

**Affiliations:** 1 CEDOC – Chronic Diseases Research Center, NOVA Medical School, NOVA University, Lisboa, Portugal; 2 Faculty of Pharmacy, University of Coimbra, Coimbra, Portugal

**Keywords:** Novel antimicrobials, Anti-biofilm drugs, ESKAPE pathogens, Non-steroidal anti-inflamatory drugs, Adjuvant therapy, NSAIDs

## Abstract

**Introduction:**

Multiple-drug resistant bacteria are emerging exponentially in healthcare units, threatening public health and requiring novel therapeutic approaches. In 2017, World Health Organization published a list that frames antimicrobial resistant bacteria into priority levels for research of novel drugs to fight them.

**Methods & Materials:**

Antimicrobial resistant ESKAPE (*Enterococcus faecium*, *Staphylococcus aureus*, *Klebsiella pneumoniae*, *Acinetobacter baumannii*, *Pseudomonas aeruginosa*, *Enterobacter* sp.) and *Enterococcus faecalis* and *Escherichia coli* pathogens are present in this list. Representative isolates of each species were used to test the Antibacterial and anti-biofilm formation activities of Etodolac (a Non-Steroidal Anti-Inflammatory Drug, NSAID) at 10 and 1 mM using a broth microdilution technique.

**Results & Discussion:**

Statistically significant (p< 0,05) results were observed against all tested gram-positives, particularly anti-biofilm activity against *E. faecium*. Etodolac had an almost null influence on tested gram-negatives, with the exception of one *A. baumannii* clinical isolate regarding biofilm formation inhibition. Observed differences deserve further analysis and prospection of the involved mechanisms, to unravel possible novel bacterial targets for drug development. Similar work with other NSAID’s may also be worth exploring to ascertain novel therapeutic applications for these drugs, particularly regarding biofilm formation inhibition, *per si* or as adjuvants of current antibiotherapy, mainly against gram-positives, as suggested by present work.

**Conclusion:**

Already approved drugs in terms of pharmacokinetics and safety may deploy faster solutions for antimicrobial therapy against priority pathogens. Current work intends to bring attention to that possibility, particularly regarding NSAIDs, anti-biofilm formation and top priority pathogens.

## 1 INTRODUCTION

Antimicrobials appeared in the beginning of the 20^th^ century and changed the world. Since its introduction in the clinical practice, life expectancy at birth almost doubled from 47 to 79 years. For the first time in Human history, Medicine could offer a solution for cholera, diphtheria, pneumonia, typhoid fever, plague, tuberculosis, typhus, syphilis and several other infectious diseases [1]. However, with a galloping increase of its use since the very beginning, physicians rapidly assisted to an also galloping emergence and dissemination of different types of Antimicrobial Resistance (AMR), especially among bacteria. Currently, AMR is considered one of the 21^st^ century major concerns, particularly multiple-Drug Resistance (MDR) and the even more concerning Extensively Drug-Resistance (XDR) and Pan Drug-Resistance (PDR) [2]. The “antibiotic era” is now threatened and scientific community is already announcing the rise of a fateful “post-antibiotic era”, with no options to treat infections [1, 3].

Available antimicrobial agents are no longer enough to treat some infections caused by MDR, XDR or PDR strains, and numbers are rising [3]. Considering the urgency of the problem, the World Health Organization (WHO) recently listed AMR bacteria into priority levels for the search, at a fast-paced rhythm, of novel drugs to treat AMR related infections [4]. To accelerate drug development, which is (correctly) deemed to several time-consuming steps, drugs belonging to pharmacological classes already approved in terms of pharmacokinetics and safety (thus, saving time in the development and validation process) are now, and from a while ago, seen as possible antimicrobial or adjuvant therapeutic options. Several studies have already demonstrated antimicrobial properties in drugs from distinct pharmacological classes, with long-term use in the clinical practice for other therapeutic goals [5]. Non-steroidal anti-inflammatory drugs (NSAID) [6], antihistaminic [7], antipsychotic [8], local anesthetics [9] and cardiovascular drugs [10] are some of the non-antimicrobial pharmacological classes that already have some molecules tested for their antimicrobial activities, with relevant *in vitro* and *in vivo* results. These molecules may be of possible interest as helper agents in the future treatment of AMR related infections.

Etodolac (ET) is a NSAID with anti-inflammatory, analgesic and antipyretic properties due to a selective inhibition of cyclooxygenase (COX)-2 by reducing prostaglandin production in the host. ET long term therapy is mainly used to treat osteoarthritis, rheumatoid arthritis and ankylosing spondylitis [11]. Short term therapy is used in acute pain [12]. Other COX-2 selective inhibitors have already showed antimicrobial activity [13, 14]. We hypothesize ET can also have antimicrobial activity and may represent a treatment alternative in the future.

ESKAPE pathogens – *Enterococcus faecium*, *Staphylococcus aureus*, *Klebsiella pneumoniae*, *Acinetobacter baumannii*, *Pseudomonas aeruginosa* and *Enterobacter* species – is a recent designation proposed in the literature to include the most prevalent pathogens in hospitals [15]. *Enterococcus faecalis* and *Escherichia coli* are also prevalent. All these pathogens characteristically present MDR profiles. Their ability to form biofilms also plays a significant role in its AMR feature and persistence in healthcare facilities. Altogether, these pathogens remain a constant threat to patient’s welfare and a huge financial burden to the health systems [3, 16].

In current study we explored our ET as antimicrobial or adjuvant agent hypothesis by testing its antibacterial and anti-biofilm formation ability against representatives of ESKAPE, *E. faecalis* and *E. coli* pathogens.

## 2 MATERIALS AND METHODS

### 2.1 Bacterial Isolates

Bacterial isolates used in this study were obtained from the American Type Culture Collection (ATCC) or from a Portuguese central hospital, as summarized in Table **1**. Clinical isolates were identified using VITEK MS (BioMérieux). All isolates were cultured in Mueller-Hinton (MH) agar (Himedia) and cryopreserved at -80ºC in MH broth (Himedia) supplemented with 15% glycerol (VWR).

### 2.2 ET Solutions

Being insoluble in water, ET stock solutions were prepared in 100% dimethyl sulfoxide (DMSO) (Merck) at room temperature, with a final concentration of 500 mM (maximum achievable ET concentration in DMSO without precipitation). Working solutions (400 and 40 mM) were obtained by dilution of stock solution in DMSO and sterile filtered (0.22 µm pore size) before use. For the antibacterial and anti-biofilm challenge experiments these working solutions were primary diluted to 20 and 2 mM in MH broth. No ET precipitation was observed under these conditions. All solutions were prepared 30 minutes prior to the experiments to prevent unspecific degradation of the compound.

### 2.3 Determination of ET Inhibitory Activity Against Bacterial Growth

ET inhibitory activity against bacterial growth was determined using a broth dilution technique in sterile, flat bottom, 96 wells microplates [17]. Briefly, ET working solutions were diluted in MH broth to a final concentration of 20 and 2 mM, respectively, and 100 µl of each final solution was distributed in 18 wells. To determine the influence of ET diluent (DMSO) in bacterial growth, 18 wells were also prepared with 100 µl MH broth supplemented with DMSO. The remaining wells were used as positive (MH broth inoculated with the test strains) and blank controls. Fresh cultures of each tested strain were suspended in 1 McFarland turbidity sterile saline and diluted to 1:1000 in MH broth 5 minutes prior to inoculation. 100 µl of the diluted bacterial suspensions was inoculated in the corresponding wells, thus obtaining an ET final concentration of 10 and 1 mM. Microplates were incubated at 37ºC for 20±4 hours. Turbidity was measured by Optical Density (OD) at 600 nm using a spectrophotometer (Shimadzu). Experiments were performed in triplicate.

### 2.4 Determination of ET Inhibitory Activity Against Biofilm Formation

Inhibition of biofilm formation by ET was determined as described elsewhere [17]. Briefly, after total bacterial growth inhibition determination, planktonic cells were removed and wells were stained with 1% crystal violet (v/v) for 20 minutes. Microplates were thoroughly rinsed to remove excess dye. The dye remaining inside the biofilm cells were solubilized with 100 µl 70% ethanol and dye density was measured at 600 nm with a spectrophotometer (Shimadzu). Experiments were also performed in triplicate.

### 2.5 Statistical Analysis

Statistical analysis was performed using SPSS^®^ version 21 (Chicago, USA). Normality of each group of results (planktonic/biofilm growth with DMSO, ET 1 mM and ET 10 mM – 18 reads/condition, in triplicate) was confirmed by Kolmogorov-Smirnov test. Mean values and standard deviations for each experiment were determined.

For normal distributions, Levene test was used to evaluate variance equality (p-value>0,05) prior to ANOVA analysis. Post-hoc tests (Games-Howell or Bonferroni) were performed whenever ANOVA analysis proved that the differences between the means of the 3 tested conditions were statistically significant (p-value<0,05). In post-hoc tests, differences in the means of each tested pair of conditions were considered statistically significant when tests returned p-values<0,05.

For non-normal distributions, Kruskall-Wallis analysis was used, followed by post-hoc multi-comparison tests, with similar statistical interpretation.

## 3 RESULTS

Inhibition of Planktonic Growing cultures (IPG) and Inhibition of Biofilm Formation (IBF) by ET against ESKAPE and no-ESKAPE tested isolates results were normalized to control (growth in DMSO only) and are presented in Figs. (**1** and **2**). Respective statistical analysis is presented in Table **2**.

ATCC – American Type Culture Collection; n.a. – not applicable (ANOVA p-value>0.05 – the difference between the means of the 3 tested conditions were not statistically significant); * - result from Games-Howell test; º – result from Bonferroni test; “ – results from multiple comparison analysis of non-normal distribution; p-values in bold are statistically significant (<0.05).

**Fig. (1):**
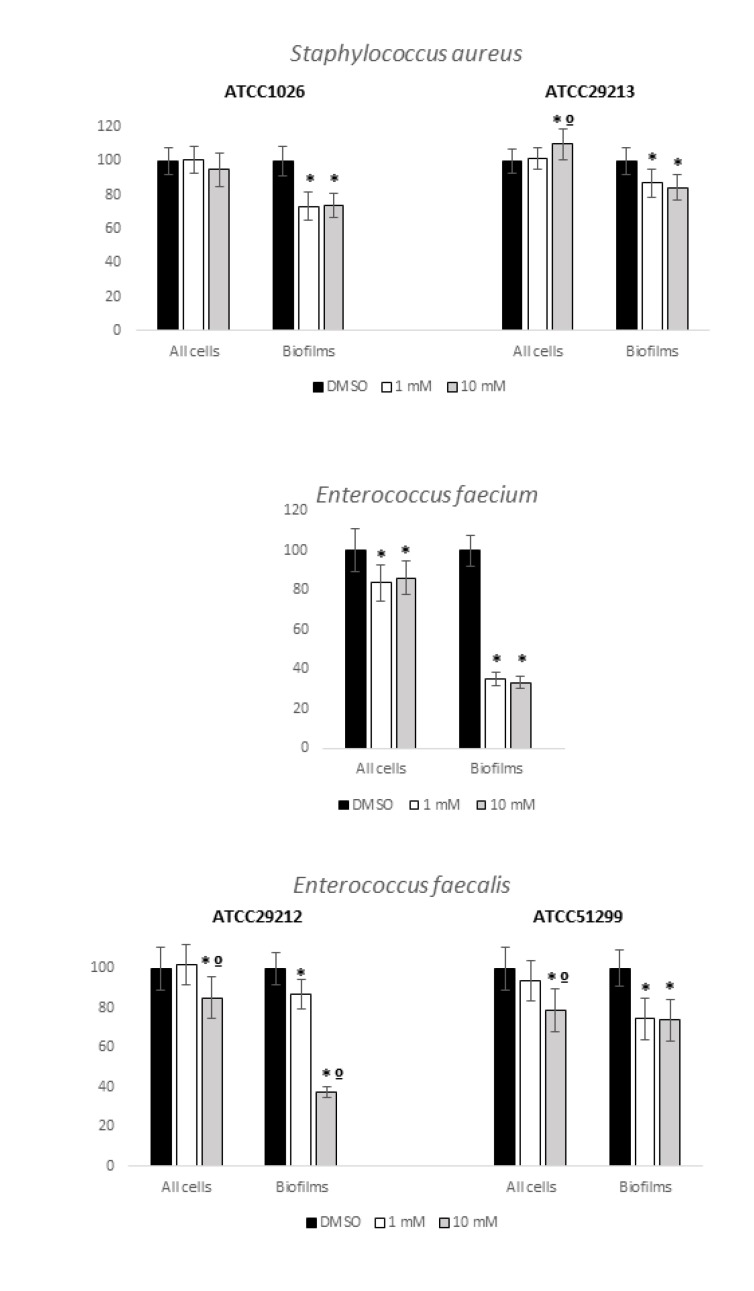
Comparison of etodolac (ET) 1mM and 10mM ability to inhibit planktonic growth (all cells) and biofilm formation (biofilms) in gram-positives *Staphylococcus aureus*, *Enterococcus faecium* and *Enterococcus faecalis*. Results are normalized to ET diluent dimethyl sulfoxide (DMSO). * - statistical significant difference (p<0,05 on multiple comparison analysis) between ET and DMSO; º - statistical significant difference (p<0,05 on multiple comparison analysis) between ET 1mM and ET 10 mM.

Overall, ET presented better IPG and IBF against gram positives than against gram-negatives, particularly *E. faecium* (Fig. **1**). The tested clinical isolate evidenced a prominent statistically significant decrease of around 70% in biofilm formation when challenged with both tested ET concentrations. A decrease in planktonic growth was also observed, with statistical significance, but not so prominent as in biofilm formation. Statistically significant results were also observed regarding IBF in the tested *S. aureus* isolates (both ATCC strains) and *E. faecalis* (both clinical isolates) but not as evident as in *E. faecium* (Fig. **1**). Regarding planktonic growth, it is relevant to highlight that one of the two tested *S. aureus* (ATCC29213) grew better with ET than with DMSO only (Fig. **1**). Regardless this particular observation, the performed tests of ET activity against the studied gram-positive isolates, although with different magnitudes, were similar in their overall results and thus suggestive of a possible general inhibitory activity of ET against gram-positives that deserves being explored, particularly regarding biofilm formation.

**Fig. (2):**
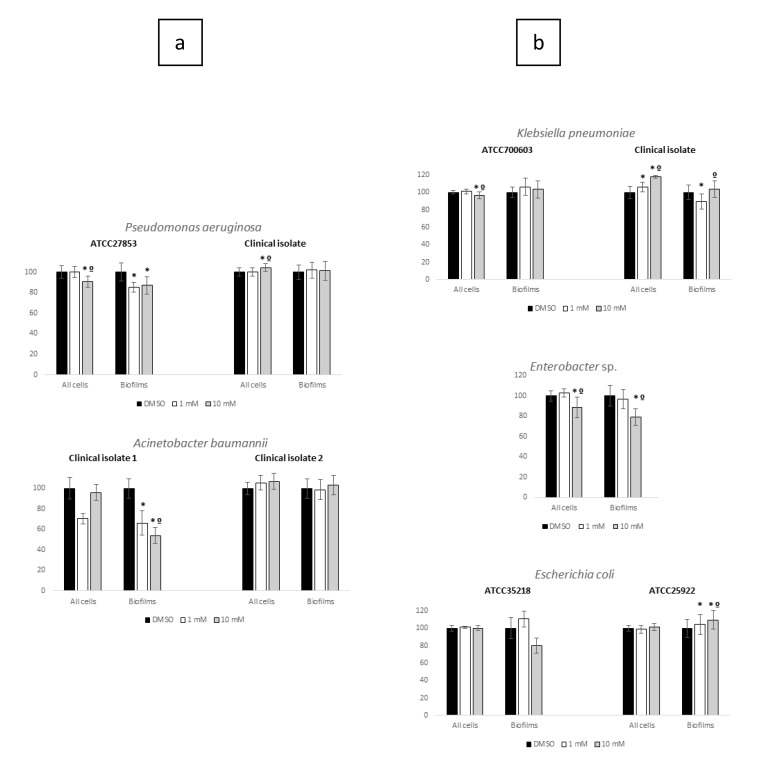
Comparison of etodolac (ET) 1mM and 10mM ability to inhibit planktonic growth (all cells) and biofim formation (biofilms) in gram-negatives Pseudomonadaceae *Pseudomonas aeruginosa* and *Acinetobacter baumannii* (**a**) and Enterobacteriaceae *Klebsiella pneumoniae*, *Enterobacter* sp. and *Escherichia coli* (**b**). Results are normalized to ET diluent dimethyl sulfoxide (DMSO). * - statistical significant difference (p<0,05 on multiple comparison analysis) between ET and DMSO; º – statistical significant difference (p<0,05 on multiple comparison analysis) between ET 1mM and ET 10 mM.

Considering the tested gram-negatives, apart from the practical ESKAPE classification, the species used in this study were grouped according to its phylogeny in Pseudomonadaceae family regarding *P. aeruginosa* (one ATCC, one clinical isolate) and *A. baumannii* (two clinical isolates), both belonging to the ESKAPE group, and in Enterobacteriaceae family including the ESKAPE *K. pneumoniae* (one ATCC, one clinical isolate) and *Enterobacter* sp. (one clinical isolate), and the no-ESKAPE *E. coli* (two ATCC isolates). Results of ET IPC and IBF challenge experiments are presented in (Fig. **2**). Contrarily to what was observed in the tested gram-positives, ET influence in IPG and IBF were diverse within gram-negative group, and even within each phylogenic family and species. Indeed, within Pseudomonadaceae members, ET IPG and IBF were scarce on both tested *P. aeruginosa* isolates, while tested *A. baumannii* clinical isolates presented divergent behaviors. Both concentrations of ET diminished *A. baumannii* clinical isolate 1 biofilm formation by around 50%, while scarcely influencing biofilm formation in *A. baumannii* clinical isolate 2 (Fig. **2a**).

Bacterial and biofilm growth of the tested Enterobacteriaceae were also little influenced by ET and in some cases were even slightly greater, particularly regarding *K. pneumoniae* clinical isolate bacterial growth and *E. coli* ATCC25922 biofilm formation (Fig. **2b**). In sum, ET showed little and inconsistent IGP and IBF activity against representatives of two prominent gram-negatives phylogenetic families and members of ESKAPE group, leading to the assumption that this compound does not offer as a possible alternative for future treatment strategies of infections caused by AMR gram-negatives. However, further studies are required to confirm this assumption.

## 4 DISCUSSION

AMR management is a top priority for WHO [4]. An estimation of 10 million deaths/year in 2050 due to untreatable infections is already established [18]. Finding novel alternatives to treat infections caused by MDR, XDR or PDR pathogens is paramount to prevent an increasingly announced involution of global health due to the rise of untreatable infections [1, 2]. Drug development is highly burdensome and time consuming. Exploring the antimicrobial potential of already approved drugs in terms of pharmacokinetics and safety greatly diminishes this burden, releases budget to other areas of drug development and, more importantly, accelerates the availability of novel strategies to fight MDR, XDR and PDR infections.

ET is a COX-2 inhibitor NSAID, a pharmacological class that already evidenced antimicrobial activity in previous studies [13, 14]. Moreover, NSAIDs are sometimes used in combination to treat inflammatory processes caused by bacterial infections in mucosal surfaces, but this approach has been questioned as suppressing inflammation may diminish the immune system capacity to respond to bacterial infections [19]. Current study tested the IPG and IBF ability of ET against isolates of the ESKAPE group, that contains the currently most concerning pathogens in the hospital setting, as well as *E. faecalis* and *E. coli*, also worrisome in terms of AMR and infection control in healthcare facilities [4]. Observed IPG and IBF, although generally sparse, was quite different between gram-positives and gram-negatives. Antibacterial activity was mainly observed in the first group, especially against biofilm formation, which was diminished in all gram-positives under the influence of ET at 1 mM or 10 mM, and particularly the tested *E. faecium* isolate.Whilst not explored in current study, the observed differences in biofilm formation between gram-positives and gram-negatives may be related to their different cell walls, since biofilm formation is highly dependent of several biochemical processes occurring in bacterial cell walls [20, 21]. NSAIDs are lipophilic and known to inhibit diverse cell membrane associated mechanisms in eukaryotic cells [22]. Similar influence may occur in prokaryotic cell walls and/or membranes associated biochemical processes, including those related to biofilm formation and/or bacterial growth, which could explain the observed results in this study. On the other hand, ET most relevant mechanism of action occurs in the cytoplasm, thus requiring its entrance in the cell. The additional outer membrane of gram-negative bacteria and its different cell wall composition diminish cell penetration [23]. We can also speculate that the observed lower IPG and IBF activity of ET against the tested gram-negative isolates could be related to its lower penetration in gram-negative cells. However, this possibility does not explain the increased bacterial growth observed in some gram-negatives. Also, in the face of current results and considering the above mentioned NSAID use in mucosal inflammation caused by bacterial infection [19], we may also consider that ET antibacterial/anti-biofilm activity occurs not *via* the traditionally known NSAIDs mechanism of action, but by a yet unknown process. Considering the evidenced results in present study, we believe that the specific mechanism of ET antibacterial and especially its anti-biofilm action in gram-positives deserve further exploration since it may provide important insights into novel NSAIDs therapeutic applications and novel bacterial targets for drug development.

High-levels of MDR among gram-positive clinical isolates are a major concern worldwide, being responsible for 2 million infections and 23000 deaths/year in developed countries, particularly Vancomycin-Resistant Enterococci (VRE), Methicillin-Resistant *S. Aureus* (MRSA), Vancomycin-Intermediate *S. aureus* (VISA) and Vancomycin-Resistant *S. aureus* (VRSA) [1]. The “Global priority list of antibiotic-resistant bacteria to guide research, discovery, and development of new antibiotics” highlights the urgency of discovering novel drugs to specifically treat these highly concerning MDR pathogens [4]. Current study evidenced a significant anti-biofilm activity of ET against all tested gram-positives, but especially against the Enterococci *E. faecium*, which is extremely difficult to treat [24]. These results may envision a possible use of ET as a helper agent in the treatment of MDR *E. faecium* infections. Additional studies to explore this possibility should also be undertaken.

Regarding gram-negatives, despite the observed overall poor influence of ET in IPG and IBF, the compound showed some IPG and IBF activity against one of the two tested *A. baumanii* isolates. Variability on antimicrobial resistance and virulence in clinical isolates is known and associated to poorer outcomes [2, 25]. *A. baumannii* is not an exception and its increasingly variable rates of drug resistance in different countries, particularly against carbapenems, are known and contribute to its inclusion in the ESKAPE group and in WHO critical priority level for research of new antibiotics [4]. Observed differences in tested *A. baumannii* isolates could be associated with differences in their resistance or virulence features, but the authors did not have the opportunity to test this. Further studies with higher numbers of isolates from this species should also be carried out, to clarify current results, as well as its relation with AMR and virulence.

Finally, and of paramount note, is the fact that ET demonstrated to be more efficient in inhibiting biofilm formation, which is already demonstrated to play a major role in AMR [16, 20, 21]. Bacteria cells in biofilms are more difficult to eliminate. This is highly relevant as currently there are no drugs in clinical use that specifically target biofilms albeit the already acknowledged fact that anti-biofilm compounds may not have great antimicrobial activity but can render bacteria cells to a planktonic growth state which make them more susceptible to conventional antimicrobials and clearance by the immune system [26]. The observed little antimicrobial but greater anti-biofilm activities of ET against representatives of highly concerning pathogens, particularly the worrisome *E. faecium*, deserve attention from the scientific community and more studies should be carried out, primarily with bigger samples to confirm results, and secondly to investigate the involved mechanisms of ET and other related NSAIDs anti-biofilm activity. We believe that the acquired knowledge will be helpful to underpin novel bacterial drug targets and novel therapeutic applications of currently available non-antimicrobial drugs that can act as helper agents in MDR infections particularly related to biofilm formation.

## CONCLUSION

Preventing the emergence of the already announced post-antimicrobial era of untreatable infections due to the lack of therapeutic options is mandatory. Already approved drugs regarding pharmacokinetics and safety circumvent years of drug development workflow and expenses. Fast deployment of easily available novel antimicrobial treatment strategies and particularly novel anti-biofilm formation drugs are urgently needed for the demand highlighted by WHO regarding top priority pathogens management worldwide, like *E. faecium*. We believe this study opens up possible windows of opportunity that deserve being explored.

## Figures and Tables

**Table 1:** Bacterial isolates tested against etodolac.

Bacterial Species	Isolate	Classification^1^	**Phylogeny (Family)**
**Gram positives**			
*Staphylococcus aureus*	ATCC 1026	ESKAPE	Staphylococcaceae
*Staphylococcus aureus*	ATCC 29213	ESKAPE	
*Enterococcus faecium*	clinical isolate	ESKAPE	Enterococcaceae
*Enterococcus faecalis*	ATCC 29212	no-ESKAPE	
*Enterococcus faecalis*	ATCC 51299	no-ESKAPE	
**Gram negatives**			
*Pseudomonas aeruginosa*	ATCC 27853	ESKAPE	Pseudomonadaceae
*Pseudomonas aeruginosa*	clinical isolate	ESKAPE	
*Acinetobacter baumannii*	clinical isolate 1	ESKAPE	
*Acinetobacter baumannii*	clinical isolate 2	ESKAPE	
*Klebsiella pneumoniae*	ATCC 700603	ESKAPE	Enterobacteriaceae
*Klebsiella pneumoniae*	clinical isolate	ESKAPE	
*Enterobacter* sp.	clinical isolate	ESKAPE	
*Escherichia coli*	ATCC 25922	no-ESKAPE	
*Escherichia coli*	ATCC 35218	no-ESKAPE	

^1^ according to reference 15; ATCC – American Type Culture Collection.

**Table 2:** Results of the statistical comparison between the effect of etodolac (ET) on the inhibition of planktonic growth and inhibition of biofilm formation in selected ESKAPE and no-ESKAPE pathogens, comparatively to the results obtained with ET diluent dimethyl sulfoxide (DMSO), using post-hoc multiple comparison analysis after ANOVA analysis in all conditions, except for *S. aureus* ATCC 29213, that revealed a non-normal distribution on the Kolmogorov-Smirnov test and thus its mean results comparison were analyzed *via* Kruskall-Wallis prior to post-hoc multiple comparison.

**Bacterial Species**	**Isolate**	**Inhibition of Planktonic Growth**			**Inhibition of Biofilm Formation**		
		(Multiple Comparison *P*-Value)			(Multiple Comparison *P*-Value)		
		**DMSO – ET 1 mM**	**DMSO – ET 10 mM**	**ET 1 mM – ET10 mM**	**DMSO – ET 1 mM**	**DMSO – ET 10 mM**	**ET 1 mM – ET10 mM**
**Gram positives**							
*Staphylococcus aureus*	ATCC 1026	n.a.	n.a.	n.a.	0.000*	0.000*	1.000*
*Staphylococcus aureus*	ATCC 29213	1.000”	**0.005”**	**0.029”**	**0.000º**	**0.000º**	0.990º
*Enterococcus faecium*	clinical isolate	0.001*	0.001*	0.819	0.000*	0.000*	0.309*
*Enterococcus faecalis*	ATCC 29212	1.000º	**0.001º**	**0.000º**	**0.000º**	**0.000º**	**0.000º**
*Enterococcus faecalis*	ATCC 51299	1.000º	**0.000º**	**0.000º**	0.000*	0.000*	0.771*
**Gram negatives**							
*Pseudomonas aeruginosa*	ATCC27853	1.000º	**0.000º**	**0.000º**	**0.000º**	**0.000º**	1.000º
*Pseudomonas aeruginosa*	clinical isolate	1.000º	**0.013º**	**0.029º**	n.a.	n.a.	n.a.
*Acinetobacter baumannii*	clinical isolate 1	n.a.	n.a.	n.a.	0.000*	0.000*	0.000*
*Acinetobacter baumannii*	clinical isolate 2	n.a.	n.a.	n.a.	n.a.	n.a.	n.a.
*Klebsiella pneumoniae*	ATCC 700603	0.369*	0.018*	0.003*	n.a.	n.a.	n.a.
*Klebsiella pneumoniae*	clinical isolate	0.004*	0.000*	0.000*	**0.006º**	0.781º	**0.000º**
*Enterobacter* sp.	clinical isolate	0.239*	0.001*	0.000*	1.000º	**0.000º**	**0.000º**
*Escherichia coli*	ATCC 25922	n.a.	n.a.	n.a.	n.a.	n.a.	n.a.
*Escherichia coli*	ATCC 35218	n.a.	n.a.	n.a.	**0.022º**	**0.000º**	**0.000º**
